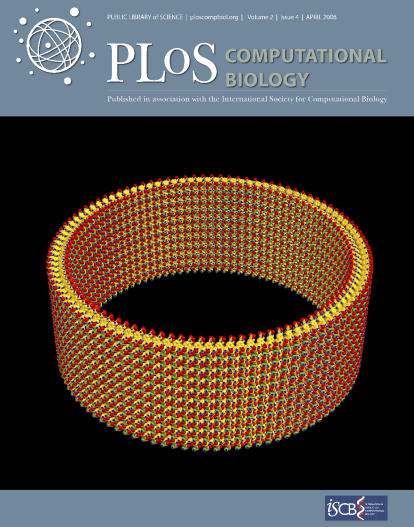# One Year of *PLoS Computational Biology*

**DOI:** 10.1371/journal.pcbi.0020111

**Published:** 2006-08-25

**Authors:** Philip E Bourne

The June 2006 issue of *PLoS Computational Biology* marked one year of publication of the journal. While it is too early to formally assess the impact of the journal, it is worth reflecting on what has been achieved in the first year of publication. Twelve monthly issues actually reflect eighteen months of submissions, given that we have been considering papers since January of 2005. At the end of June 2006, 631 research articles have been submitted to the journal, 110 have been published, and 64 (48 new submissions and 16 revisions) are currently under review. We estimate that our acceptance rate is currently about 30%, increasing in recent months from less than 20% as authors become more familiar with the expectations of the journal and do not submit papers that have little chance of being published. We are now publishing about 15 research articles per month. Accompanying these research articles over the year have been three Editorials, six Reviews, and five Perspectives, and the Education section just published its first tutorial. Time for review averages 13.2 days and for acceptance to publication is five to six weeks, although authors have the option of having their manuscripts posted as soon as they are accepted. The journal is published in association with the International Society for Computational Biology (ISCB), an important relationship whereby one author of each accepted paper gets a free one-year membership to the Society. Articles about ISCB activities are also a regular feature in the journal. As the relationship between PLoS and ISCB matures in the coming years, we hope that this will provide a stimulus for other scholarly societies to explore and adopt open access publishing.

Submissions have been received from 41 countries (based on the location of the corresponding author). The top six countries submitting articles are the US (49.1%), the UK (5.1%), Japan and India (3.3% each), Netherlands (3.0%), and Israel (2.5%).

Interest in the journal can be gauged by the number of people who have signed up to receive an electronic alert of journal contents (eTOC) and by the number of downloads of journal articles. Currently 7,125 people are signed up to receive eTOCs, a number that grows by several hundred each month. Since the launch, there have been more than 250,000 article downloads, comprising 200,019 research articles, 23,408 Editorials, 14,331 Perspectives, 13,869 Reviews, and a number of hits for the Education Column and for Message from the ISCB. The top ten papers downloaded thus far are shown in Table 1.

**Table 1:**
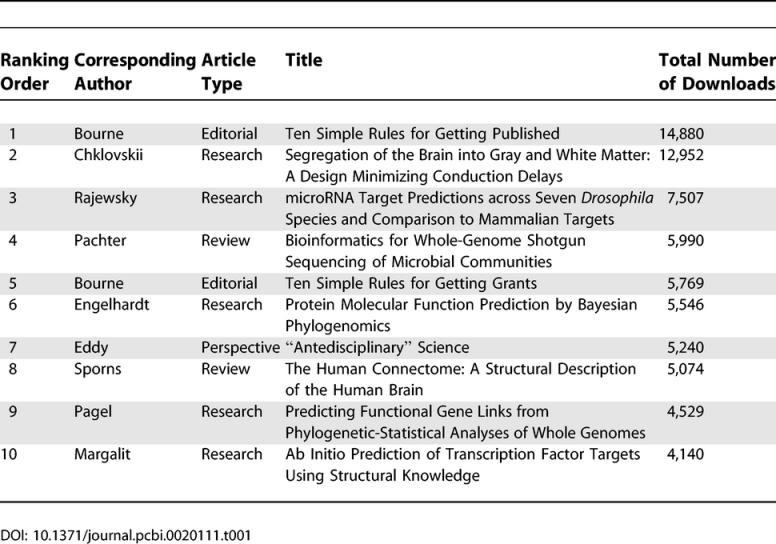
Top Ten Papers Downloaded from *PLoS Computational Biology* in Its First Year

Two of the top ten papers are in the area of neuroscience, and two others form part of the ongoing “Ten Rules” series of Editorials to aid our less experienced readers (see Table 1). Of the remainder, one is a Perspective discussing team versus individual science and the rest are in the realm of computational molecular biology. Demographics of materials downloaded show North America and Europe account for 60%–70% of the usage of the site, but there is significant usage from the developing world, a testament to open access.

Overall, we are well on the way to meeting the original editorial goal of the journal—to establish a high-quality knowledge resource serving a community interested in advancing our understanding of living systems through the use of computational techniques. While reported advances have been predominantly at the molecular level, there is a growing body of work being submitted that covers different levels of biological organization. This reflects our goal to publish great work involving computational analyses on all biological scales. We want to make connections between researchers who are using conceptually related approaches to tackle diverse issues in biology.

To put this goal in perspective, consider that in one year we have explored the RNA silencing pathway in issue 1(2), designed a nanotube using naturally occurring protein building blocks in issue 2(4), modeled the transition to quorum sensing in a population of *Agrobacterium* in issue 1(4), and understood more about the role of mechanical factors in the morphology of the primate cerebral cortex in issue 2(3), to name but a few articles. Not bad for the first year.

We aspire to have *PLoS Computational Biology* develop into an exemplar open access community journal which will provide a model for scholarly publishing of the future. The publication fee for the journal has recently increased from US$1,500 to US$2,000, and, along with the growth of the journal (in terms of submissions and published articles), the journal is moving steadily along a path towards financial sustainability. This is good news, and at the same time PLoS retains a fee waiver policy for those authors with insufficient funds, so money is never a factor in disseminating good science.

Over the course of the past year, our monthly submissions have increased to a record 55 in June. We are all tremendously gratified to see this strong community response, and it should be remembered that this support has been offered in the absence of an impact factor (perhaps not a good indicator for an open access journal, but that is another Editorial). That we have been able to maintain an efficient editorial and publishing service in the face of this growth is testament to the tremendous efforts of our Editorial Board. My thanks go to them, and also to the PLoS staff, notably Catherine Nancarrow, Emily Stevenson, and Mark Patterson who really are the ones who keep it all on track.

Join us in our second year by getting your work published in this fast-growing and diverse journal, which we hope will make those of us involved in Computational Biology proud of our collective efforts in this rapidly evolving field.

## 

**Figure:**